# Spherical Carbon Derived from Sustainable Sources and Decorated with Silver Nanoparticles as a Catalyst for Hydrogen Release

**DOI:** 10.3390/ma18214912

**Published:** 2025-10-27

**Authors:** Erik Biehler, Tarek M. Abdel-Fattah

**Affiliations:** Applied Research Center, Thomas Jefferson National Accelerator Facility, Department of Biology, Chemistry and Environmental Science, Christopher Newport University, Newport News, VA 23606, USA; erik.biehler@cnu.edu

**Keywords:** hydrogen, catalysis, sustainable materials, nano-materials

## Abstract

The reliance on carbon-based fuels remains a major contributor to greenhouse gas emissions, emphasizing the need for sustainable alternatives such as hydrogen. Sodium borohydride (NaBH_4_), with a hydrogen content of 10.6 wt%, is a promising chemical hydrogen storage material capable of releasing four moles of H_2_ per mole through hydrolysis; however, effective catalysts are essential for practical implementation. In this study, silver nanoparticles supported on glucose-derived carbon microspheres (AgSC) were synthesized and evaluated for catalytic NaBH_4_ hydrolysis. Structural characterization (XRD, TEM, SEM, EDS) confirmed the uniform dispersion of metallic silver nanoparticles on the carbon support with no detectable Ag_2_O phase. AgSC exhibited superior catalytic activity compared to unsupported Ag or bare carbon, achieving the highest hydrogen generation under neutral pH, elevated temperatures, and 835 µmol NaBH_4_. The catalyst displayed an activation energy of 54 kJ mol^−1^, turnover numbers (TONs) of 1.4 × 10^5^–1.9 × 10^5^**,** and turnover frequencies (TOFs) of 7.1 × 10^4^–9.3 × 10^4^ h^−1^, demonstrating efficient utilization of active sites. pH-dependent studies revealed optimal hydrogen yield under neutral conditions, while acidic and basic media reduced performance due to surface poisoning and BH_4_^−^ stabilization, respectively. Reusability tests showed only ~5% activity loss after five cycles. These findings establish AgSC as a stable, efficient, and recyclable catalyst for on-demand hydrogen generation, supporting sustainable clean fuel technologies.

## 1. Introduction

It is no secret that Earth’s fossil fuel resources are dwindling. Human populations continue to grow each year, with some predictions showing upwards of 10 billion people by the year 2040 [1]. As the population grows, so does the amount of fossil fuels consumed, as well as the associated greenhouse gas (GHG) emissions [2,3]. Thus, a great deal of research has focused on the search for a renewable energy source. Current renewable energy sources include solar [4], wind [5], tidal [6], and geothermal [7] energy, along with hydrogen [8] as a clean energy carrier. Each of these renewable technologies has distinct advantages and challenges; however, hydrogen stands out due to its high gravimetric energy density, abundance, and environmental compatibility. When utilized as a fuel, hydrogen produces only water as a by-product upon combustion (Equation (1)), making it a key enabler of future carbon-neutral energy systems.

(1) $${2H}_{2}+O_{2}\to 2H_{2}O$$

One of the major drawbacks preventing the widespread adoption of hydrogen fuel technologies is the challenge associated with safe and efficient hydrogen storage. Current approaches include gaseous compression [9], cryogenic liquefaction [10], chemical storage in metal hydrides [11,12], and physisorption or chemisorption on porous materials [13,14]. Although each technique offers advantages, they are generally limited by high costs, energy-intensive processes, safety risks, and limited reversibility. Notably, the latter two strategies aim to mitigate some of the hazards associated with high-pressure or cryogenic methods, yet they still face issues of scalability and long-term stability.

Rather than storing a large volume of combustible gas or using subzero temperatures, metal hydrides can release stored hydrogen steadily under ambient conditions [11]. Sodium borohydride is one such metal hydride that is of great interest since it contains 10.8% hydrogen by weight and reacts readily in water at room temperature (Equation (2)) [11]. A major drawback of this reaction is that it proceeds slowly and requires the use of a catalyst to optimize it.

NaBH_4_ + 2H_2_O → NaBO_2_ + 4H_2_ (ΔH < 0) (2)

Precious metals such as gold [15], silver [16], palladium [17], and platinum [18] are well known for their catalytic ability. Previous work by this team has demonstrated that these catalysts can be used to efficiently catalyze the hydrolysis of sodium borohydride [19,20,21,22]. These studies are unique in that novel composites were made by combining a precious metal catalyst with a carbon-based support. Carbon-based materials are known for their cost-effectiveness, thermal and electrical properties, hydrogen adsorption ability, and effectiveness in catalysis [23,24,25].

Nanoparticles are inherently prone to instability. In the absence of a robust support structure to anchor them, nanoparticles tend to aggregate, leading to a reduction in surface area and a subsequent decline in catalytic efficiency [26]. One approach to enhancing the catalytic performance of metal nanoparticles involves utilizing spherical carbon as a structural framework. Although spherical carbon is a relatively recent focus in research, it has demonstrated significant potential in various fields, including supercapacitors and superconductors [27,28]. Additional uses of carbon microspheres include applications in fuel cells, sensors, and catalysis [29,30,31]. The versatility of chemical activity across these diverse applications underscores the promise of spherical carbon as an adaptable and effective catalyst [32,33,34].

The morphology of nanomaterials plays a pivotal role in determining their catalytic performance, with spherical structures offering distinct advantages. One key benefit of spherical geometry is the maximization of surface area, which creates a larger interface for catalytic interactions. This increase in surface area results in a higher concentration of active sites, thereby accelerating reaction rates and improving catalytic efficiency. Furthermore, the spherical shape enhances reactant accessibility to these active sites, mitigating diffusion barriers and promoting more effective interactions with the catalyst [35]. 

An additional advantage of spherical morphology is its ability to improve mass transfer. The smooth, symmetrical structure of spheres reduces resistance to molecular movement, enabling reactants and products to navigate the catalyst surface more freely [36]. This enhanced mass transfer is especially critical in systems requiring high reaction rates to sustain dynamic processes.

In the case of borohydride hydrolysis, spherical carbon materials provide unique benefits. The size of these spheres can be adjusted to optimize catalytic performance, as sphere size directly influences reactant–catalyst interactions [37]. For instance, smaller spheres offer increased surface-to-volume ratios, enhancing active site exposure, while larger spheres create distinct diffusion pathways that can facilitate product release. The inherent symmetry of spherical carbon also ensures an even distribution of active sites, contributing to consistent reaction behavior.

Overall, the spherical morphology of carbon materials influences crucial catalytic parameters, including surface area, active site accessibility, and mass transfer efficiency. The ability to fine-tune these properties through size control makes spherical carbon an invaluable tool for advancing applications in borohydride hydrolysis.

In this study, we studied a novel spherical carbon (SC) catalyst decorated by silver nanoparticles. Dextrose was used to hydrothermally synthesize our SC support, promoting green chemistry. This composite was tested for catalytic ability under various reaction conditions.

## 2. Experimental

All materials and reagents were used as received without further purification. Analytical-grade D-(+)-dextrose (C_6_H_12_O_6_) and sodium borohydride (NaBH_4_) were obtained from Sigma-Aldrich [St. Louis, MO, USA]. Deionized (DI) water with a resistivity of 18.2 MΩ·cm was used in all synthesis and washing steps.

### 2.1. Synthesis of Spherical Carbon

Spherical carbon (SC) microspheres were synthesized via a hydrothermal carbonization method using dextrose as a carbon precursor. A 0.5 M aqueous dextrose solution was first prepared using deionized water. The solution was then transferred into Teflon-lined stainless-steel autoclave tubes (50 mL capacity), with a typical working volume of 20 mL, maintaining an approximate gas-to-liquid ratio of 3:2 to ensure sufficient internal pressure buildup during the reaction. The sealed autoclave was placed in an oven and heated at 200 °C for 4 h, allowing the dextrose to undergo dehydration, polymerization, and subsequent carbonization under elevated temperature and autogenous pressure. After completion, the system was allowed to cool naturally to room temperature. The resulting dark-brown suspension, indicative of carbon microsphere formation, was collected and subjected to centrifugation at 15,000 rpm for 30 min. The obtained solid was thoroughly washed several times with deionized water to remove unreacted species and soluble by-products.

### 2.2. Synthesis of Nanoparticles and Composites

Silver nanoparticles (AgNPs) were synthesized through the chemical reduction of silver nitrate (AgNO_3_) using sodium borohydride (NaBH_4_) as the reducing agent in the presence of sodium citrate as a stabilizing and capping agent. As in a typical synthesis process, an aqueous solution of AgNO_3_ (0.01 M) was prepared under continuous magnetic stirring. A freshly prepared NaBH_4_ solution (0.02 M) was then added dropwise to the AgNO_3_ solution at room temperature while maintaining vigorous stirring. The reaction mixture immediately turned pale yellow and gradually darkened, indicating the formation of colloidal Ag nanoparticles. During this process, sodium citrate played a dual role as both a capping and mild reducing agent, preventing particle agglomeration and promoting the formation of uniformly dispersed nanoparticles with controlled size and morphology. The colloidal suspension was stirred for an additional 30 min to ensure complete reduction of Ag^+^ ions, yielding a stable AgNP solution. To prepare the silver–carbon microsphere composite (AgSC), 100 mg of the previously synthesized carbon microspheres (SC) was dispersed in 2 mL of the AgNP aqueous suspension. The mixture was stirred thoroughly at room temperature to promote uniform adsorption and deposition of AgNPs onto the carbon surface via incipient wetness impregnation. The resulting suspension was then transferred to a glass dish and dried in an oven at 100 °C for 48 h, allowing for gradual evaporation of excess water and firm anchoring of the nanoparticles within the carbon matrix. The dried AgSC composite powder was gently ground to ensure homogeneity and stored in an airtight container prior to characterization and catalytic testing. The structural, morphological, and compositional features of the AgSC catalyst were analyzed using X-ray diffraction [P-XRD, Rigaku Miniflex II, Cu Kα X-ray, nickel filters, Rigaku, Tokyo, Japan], scanning electron microscopy [SEM, JEOL JSM-6060LV, JEOL, Akishima, Tokyo, Japan] with energy-dispersive X-ray spectroscopy [EDS, Thermo Scientific UltraDry, Thermo Fischer Scientific, Waltham, MA, USA], and transmission electron microscopy [TEM, JEM-2100F, JEOL, Akishima, Tokyo, Japan] confirming the successful deposition and uniform distribution of metallic silver nanoparticles on the carbon microsphere surface.

### 2.3. Catalysis

The catalytic performance of the AgSC nanocomposite was evaluated through a hydrogen evolution reaction using sodium borohydride (NaBH_4_) as the reducing agent. Hydrogen generation was quantified using a gravimetric water displacement system, which allowed for real-time monitoring of gas evolution. The setup consisted of a series of interconnected Buchner flasks joined by airtight plastic tubing. Each flask contained 100 mL of deionized water, with one serving as the reaction vessel in which NaBH_4_ hydrolysis occurred in the presence of the catalyst, and the other functioning as the displacement vessel for hydrogen collection. The displacement flask was fitted with a rubber stopper connected to additional tubing that directed the released hydrogen into a glass flask placed on an electronic balance [Ohaus Pioneer Balance, PA124, OHAUS, Parsippany, NJ, USA] equipped with proprietary mass-logging software [version 2.04]. The system continuously recorded the mass of displaced water at 0.25 s intervals which was subsequently converted to hydrogen volume under standard conditions. Each experimental run was repeated three times to ensure reproducibility, generating over 3000 data points per trial. This high-resolution dataset provided statistically reliable results and reflected the actual experimental measurements rather than fitted or smoothed data, ensuring precise evaluation of catalytic hydrogen generation kinetics.

The catalytic hydrolysis of sodium borohydride (NaBH_4_) was systematically investigated under varying pH, temperature, and substrate concentration to determine the optimal reaction conditions for hydrogen generation. The experiments were conducted at pH values of 6, 7, and 8, which were adjusted using 0.1 M hydrochloric acid (HCl) and 0.1 M sodium hydroxide (NaOH) to create mildly acidic, neutral, and basic environments, respectively. The influence of temperature was assessed at 273 K, 293 K, and 303 K, while the effect of NaBH_4_ concentration was studied using 635, 735, and 835 μmol of the reducing agent. In each trial, 100 μg of catalyst—corresponding to 4.63 × 10^−5^ mmol of silver (Ag)—was employed as the active material.

All reactions were carried out in a glass reactor setup under ambient pressure, using deionized water as the reaction medium. The mixtures were continuously stirred with a magnetic stir bar throughout the 2 h duration of each experiment to ensure homogeneous mixing and uniform reactant contact. In the case of temperature-controlled experiments, the reaction vessel was enclosed within an insulated jacket, which interfered with magnetic stirring; in these cases, gentle manual agitation was periodically applied to maintain dispersion. The systematic variation in parameters allowed for direct evaluation of kinetic and environmental effects on hydrogen evolution efficiency.

## 3. Results and Discussion

**Figure 1:**
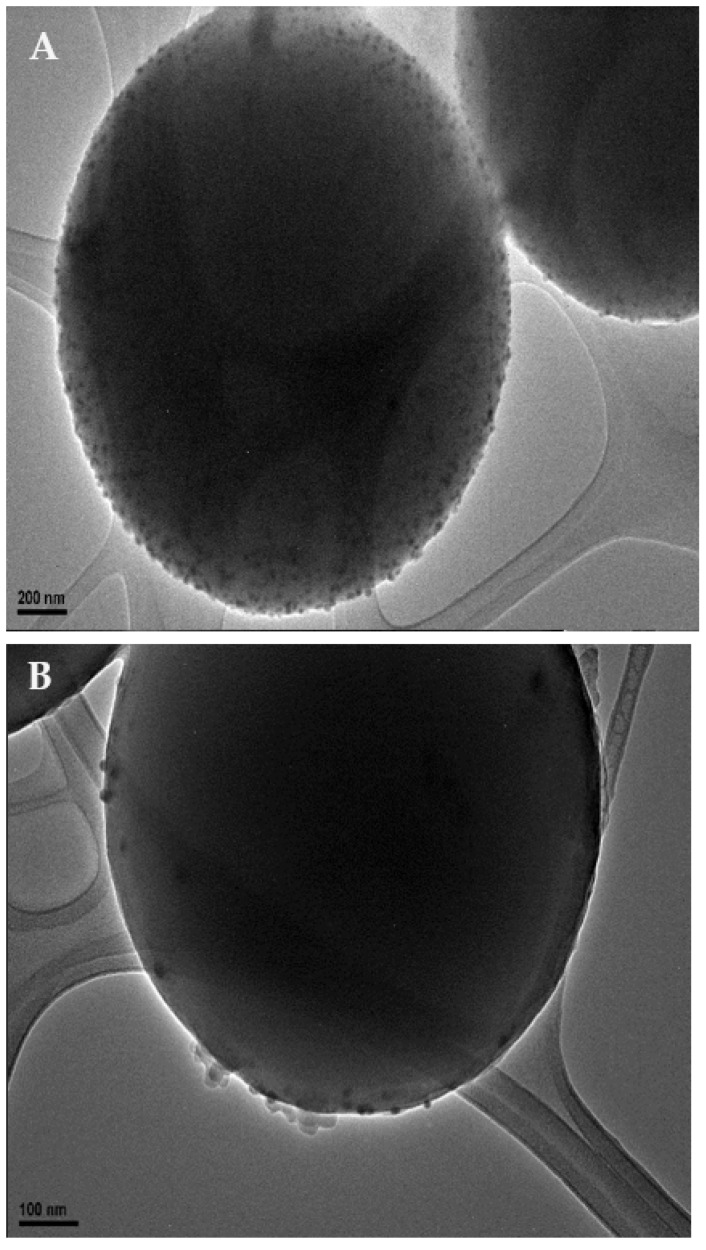

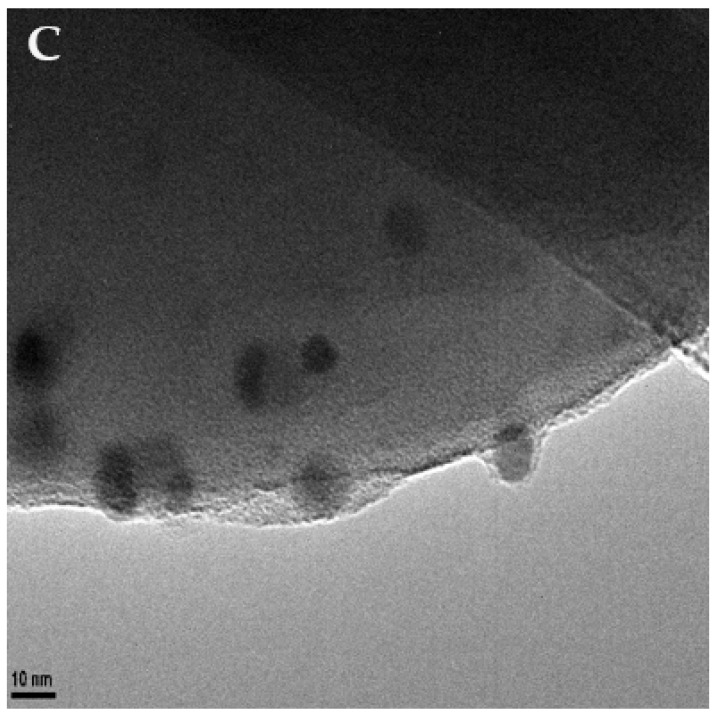
TEM images of silver nanoparticle-decorated spherical carbon. Image (**A**) depicts a scale bar of 200 nm. Image (**B**) shows a scale bar of 100 nm. Image (**C**)’s scale bar is 10 nm.

Transmission electron microscopy (TEM, Figure 1) was used to confirm the adherence of the nanoparticles upon the surface of the microspheres. TEM micrographs were also used to observe the final morphology of the nanoparticle-coated carbon microsphere composite. Further sample analysis also allowed the measurements of particles to be taken, enabling us to determine that the nanoparticles were roughly 12 ± 4 nm in diameter.

**Figure 2:**
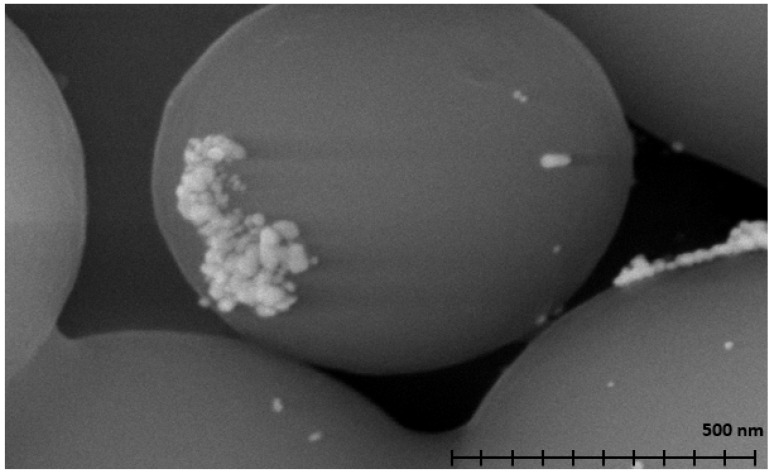
A SEM micrograph of silver-coated spherical carbon at a scale of 500 nm.

Scanning electron microscopy (SEM, Figure 2) was used to determine the size and shape of the metal nanoparticles. SEM was also used to analyze the morphology of the carbon used for microsphere synthesis and aided in the sample’s identification as spherical carbon. The BET and pore size of the material were determined to be 8 m^2^ per gram and 0.8 nm, respectively, via nitrogen adsorption [ASAP 2020 Plus microporsimeter, Norcross, GA, USA]. Energy-dispersive X-ray spectroscopy (EDX, Figure S1) was used to identify the different elements present in the sample. Very clear peaks can be seen representing carbon and oxygen, which can be attributed to the spherical carbon support interacting under regular atmospheric conditions. Two peaks can be seen around 3.0 and 3.2 keV, which are characteristic of silver. These peaks confirm that the nanoparticles seen in Figure 1 and Figure 2 are composed of silver.

**Figure 3:**
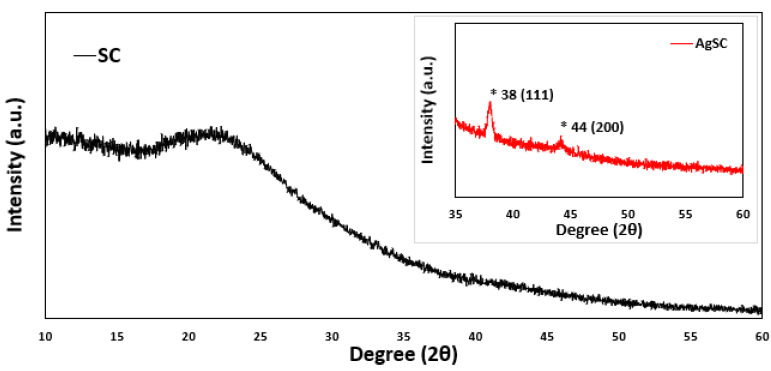
P-XRD data for the AgSC composite compared to just SC. The inset graph highlights the key peaks associated with silver which are marked with an asterisk.

The AgSC composite was characterized using PXRD and compared to SC with no silver, as seen in Figure 3. In both samples, there is an obvious broad peak seen around 20–25 degrees. These peaks are commonly seen in carbon-based materials and can be attributed to the SC material of both samples. Two peaks are present in the AgSC sample that cannot be seen in the plain SC sample at roughly 38 degrees and 44 degrees. These two peaks are likely indicative of the (111) and (200) face centered cubic (FCC) structure of silver nanoparticles with slight shifting (International Centre for Diffraction Data, ICDD 65-2871). This shifting is not significant and has been observed before as indicating a change in the lattice structure of the material [38]. These data further support the presence of silver in our samples. The average crystallite size estimated from XRD using the Scherrer equation is ~14 nm, while TEM analysis indicated an average particle size of ~12 nm. The close agreement between these two techniques supports the reliability of the measurements and suggests that the particles are largely crystalline with minimal amorphous content. Minor differences are expected: XRD reflects the size of coherent diffracting domains, which may slightly overestimate size if the particles are polycrystalline, whereas TEM provides direct imaging of overall particle dimensions, including both crystalline and amorphous regions. The 2 nm discrepancy falls within typical experimental uncertainty and indicates that the silver nanoparticles are nearly single-crystalline, with TEM and XRD giving consistent size evaluations.

### 3.1. Catalytic Studies

**Figure 4:**
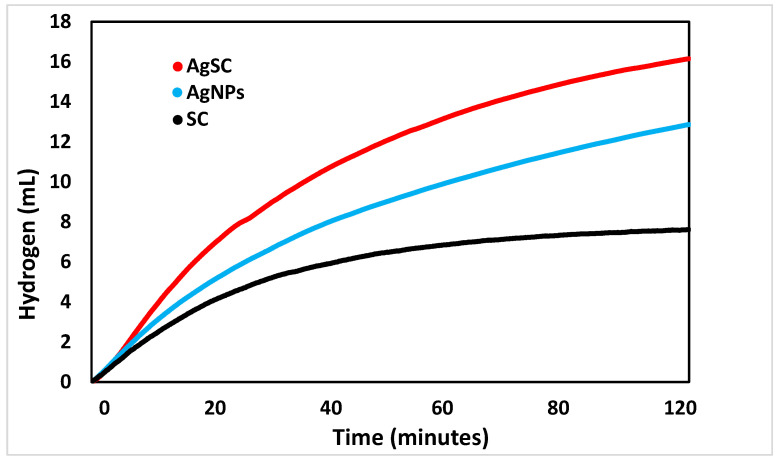
A comparison of the hydrogen volume generated over two hours by Ag nanoparticles, spherical carbon, and a composite of the two, AgSC.

The initial catalytic study compared the performance of each component of the composite—silver nanoparticles (AgNPs), spherical carbon (SC), and the combined AgSC catalyst—to assess their individual and synergistic contributions (Figure 4). The spherical carbon alone exhibited the lowest catalytic activity, generating only 7.6 ± 0.5 mL of H_2_ after 120 min. In contrast, unsupported AgNPs produced a higher volume of hydrogen, approximately 12.9 ± 0.5 mL after 120 min. The AgSC composite, however, demonstrated the highest activity, yielding 16.1 ± 0.5 mL of hydrogen under identical conditions [39]. While carbon materials have shown catalytic potential in certain reactions, their performance here is significantly lower than that of the noble metal silver. The superior activity of AgSC compared to unsupported AgNPs suggests that the carbon microsphere support effectively prevents nanoparticle agglomeration, enhances active site dispersion, and facilitates mass transfer. These combined effects likely contribute to the enhanced catalytic efficiency observed for the composite system.

The next catalytic experiment, as seen in Figure S2, tested the volume of hydrogen generated at varying mass of sodium borohydride. The lowest mass of sodium borohydride used was 635 µmol and produced the least volume of hydrogen gas, 12.4 ± 0.5 mL. The next highest volume of hydrogen produced after two hours was 15.3 ± 0.5 mL using 735 µmol of NaBH_4_. The final concentration studied was 835 µmol, which produced 16.1 ± 0.5 mL of hydrogen over the two-hour period. These results were expected as Le Chatelier’s principle states that an increase in the amount of reactant used should shift the reaction to increase the amount of product produced.

The next catalytic study we performed explored the relationship between the pH of the solution and the volume produced (Figure S3). Starting with a neutral solution pH (7), the AgSC catalyst produced approximately 16.1 ± 0.5 mL of hydrogen after two hours. To test the reaction under acidic conditions, the solution pH was lowered to 6 using hydrochloric acid (HCl 0.1M). After the two-hour trial, it was observed that less hydrogen was produced compared to under neutral conditions, with 15.1 ± 0.5 mL produced. Increasing the solution pH to 8 via sodium hydroxide (NaOH 0.1 M) also resulted in a further decrease in the volume of hydrogen produced, with only 12.8 ± 0.5 mL observed after two hours. This outcome was unexpected, as sodium borohydride is generally known to react more quickly with water in the presence of a strong acid [11]. The presence of higher concentrations of H^+^ ions under acidic conditions accelerates the hydrolysis process, which, in turn, increases the overall reaction rate [21,40,41,42,43]. In contrast, in basic environments, the higher concentration of OH− ions tends to favor the reactants, thereby slowing down the production of hydrogen (Equation (1)) [11]. Although sodium borohydride hydrolysis is intrinsically faster in acidic media, in practice, less hydrogen may be generated because system-level effects override the kinetics: rapid bubble formation under acid conditions can block mass transfer, acidic environments can dissolve or restructure catalysts leading to deactivation, and by-products such as boric acid or borates can deposit on surfaces and poison active sites; together, these factors truncate the reaction before all NaBH_4_ is consumed, explaining why experimental yields can be lower than the literature’s idealized kinetic predictions. Although these observations are unusual, similar results have also been reported in previous studies [22,42].

**Figure 5:**
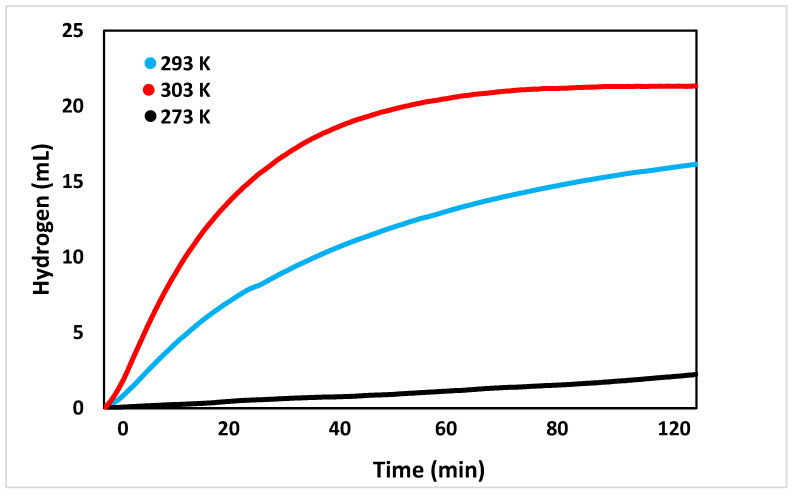
Volume of hydrogen generated over time at controlled temperatures (273, 293, 303 K).

After the pH trials were conducted, the effect of temperature on the reaction was tested, as seen in Figure 5. At first, the reaction was tested at 293 K. This temperature was observed to produce roughly 2.2 ± 0.5 mL of hydrogen after two hours. Increasing the temperature of the reaction to 293 K greatly increased the volume of hydrogen produced to 16.2 ± 0.5 mL. As suspected, increasing the reaction temperature to 303 K further increased the volume of hydrogen, with two hours of reaction time producing 21.3 ± 0.5 mL. These results indicate that the reaction is endothermic.

### 3.2. Arrhenius Plot and Determination of Activation Energy

**Figure 6:**
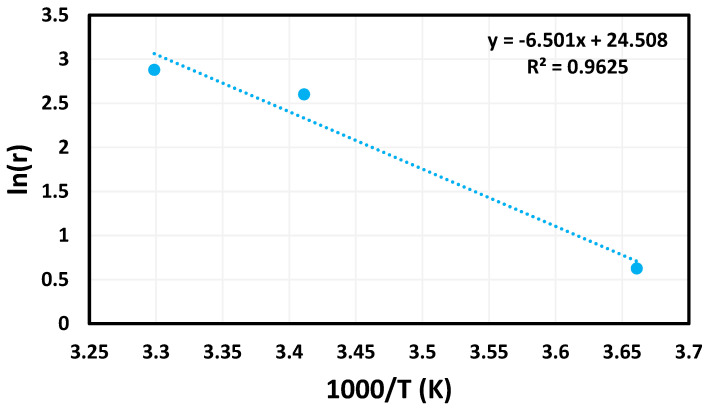
Arrhenius plot created using temperature trials.

Using the data gathered during the temperature study and the Arrhenius Equation (3), an Arrhenius plot (Figure 6) was created.

(3) $$k={Ae}^{-\frac{Ea}{RT}}$$

In the above equation, the variable *k* is representative of the rate of the reaction. The pre-exponential factor is represented by the variable *A*, while *Ea* is used for the activation energy of the reaction. Finally, *R* is the variable commonly used to represent the universal gas constant, and *T* is the temperature at which the reaction was tested. From these data, the activation energy of this reaction was determined to be 54 kJ mol^−1^.

**Figure 7:**
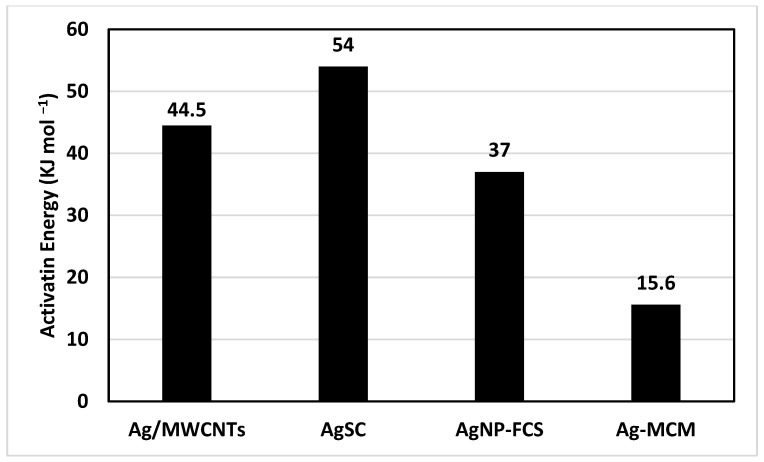
A bar chart comparing the activation energy of similar materials studied by this team.

Once the activation energy was determined, we were able to compare this catalyst to similar catalysts previously studied by this team (Figure 7). The AgSC catalyst in this study was observed to have the highest activation energy of 54 kJ mol^−1^. The next highest activation energy was 44.5 kJ mol^−1^, held by silver nanoparticles supported on multi-walled carbon nanotubes (Ag/MWCNTs) [26]. Silver nanoparticles supported on fused carbon spheres (AgNP-FCS) had a lower activation energy compared to both, at 37 kJ mol^−1^ [43]. Finally, the lowest activation energy of 15.6 kJ mol^−1^ was held by silver nanoparticles supported on mesoporous carbon (Ag-MCM) [44]. This data is interesting because all four of these composites are silver nanoparticles supported on a carbon support structure. It appears that the structure of the carbon support plays a role in the activation energy of the reaction. In another study conducted by this team, the surface area, pore size, and hydrogen uptake (wt%) were determined for a variety of different carbon materials that were used as support structures. The study showed that mesoporous carbon had the highest uptake, which may be why it had such a low activation energy compared to the other composites. Interestingly, MWCNTs had a lower uptake than spherical carbon; so, it seems that there must be more factors affecting the activation energy of this reaction. Further work should be conducted to elucidate the relationship between surface area, pore size, and hydrogen uptake and how they affect a catalyst’s ability to optimize a reaction. When compared to other catalysts used to hydrolyze NaBH_4_ (Table 1), the novel AgSC has an activation energy in the middle of the group. At 54 kJ per mol, the activation energy of the material in this study is less than that of silica sulfuric acid (17 kJ mol^−1^), cobalt chloride (17.5 kJ mol^−1^), and palladium on carbon (28 kJ mol^−1^). Of these, palladium on carbon is the most similar to our material and warrants further study as a nanomaterial on our spherical carbon support structure. Ru/Graphite, Co-B, and Ru/C all had higher activation energies at 61.1 kJ mol^−1^, 64.9 kJ mol^−1^, and 67 kJ mol^−1^, respectively. It seems from these studies that ruthenium does not represent an optimal metal for catalyzing this reaction, and it is possible that cobalt boride needs a support structure similar to our spherical carbon to lower its activation energy.

### 3.3. Kinetics Study

Table S1 summarizes the rates, turnover number (TON), and turnover frequency (TOF) for the AgSC catalyst. The TON and TOF values increase progressively from R1 to R3, reflecting the higher mass of NaBH_4_ available (636 µmol, 735 µmol, and 836 µmol, respectively). At the lowest NaBH_4_ load (R1), the total hydrogen evolved was also the lowest, resulting in the smallest TON of approximately 1.4 × 10^5^ and a TOF of about 7.1 × 10^4^ h^−1^. As the substrate amount increased in R2 and R3, both the TON and TOF improved, reaching 1.8 × 10^5^ and 8.8 × 10^4^ h^−1^ for R2, and 1.9 × 10^5^ and 9.3 × 10^4^ h^−1^ for R3, respectively. These increases reflect enhanced hydrogen generation efficiency with higher NaBH_4_ concentrations, confirming effective utilization of the available Ag surface sites in the AgSC catalyst. This trend indicates that the catalytic sites on Ag nanoparticles are capable of handling higher substrate concentrations without significant deactivation, and that hydrolysis efficiency is positively correlated with NaBH_4_ availability under the studied conditions. The relatively modest increase in the TON between R2 and R3, compared to the larger jump from R1, suggests that mass transfer effects or partial catalyst site saturation may begin to play a role at higher substrate concentrations. Overall, the results demonstrate that NaBH_4_ concentration is a key factor influencing hydrogen yield and apparent catalytic performance, though system-level effects (bubble accumulation, by-product deposition) may limit further improvements at higher loadings.

### 3.4. Reusability Study

The AgSC catalyst showed stable hydrogen generation performance over five consecutive cycles of NaBH_4_ hydrolysis, as shown in Figure S4. The initial hydrogen volume in Trial 1 was 16.1 mL, and subsequent cycles gave 15.6, 15.7, 15.4, and 15.4 mL, respectively. This corresponds to only a ~4–5% decrease in activity after five runs, indicating excellent reusability. Such stability suggests that AgNPs retain their structural integrity and surface activity without significant agglomeration or leaching during repeated use. Minor variations in hydrogen yield may arise from the slight loss of catalyst during handling or from gradual deposition of borate by-products, which can partially block active sites. Nevertheless, the consistently high hydrogen volumes across cycles demonstrate that AgNPs are robust catalysts for NaBH_4_ hydrolysis, with strong potential for practical applications where reusability is a critical requirement.

### 3.5. Proposed Mechanism of NaBH_4_ Hydrolysis over AgSC

**Scheme 1:**
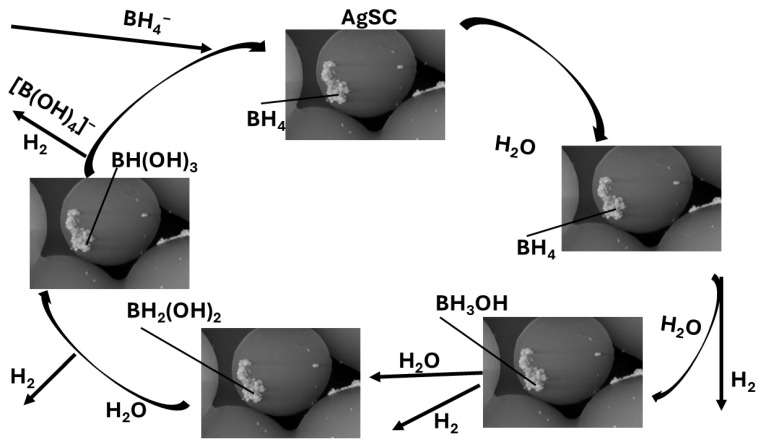
This team’s proposed mechanism of AgSC-mediated hydrolysis of NaBH_4_.

The proposed mechanism illustrated in Scheme 1 for the hydrolysis of sodium borohydride (NaBH_4_) over silver-supported carbon (AgSC) catalysts is strongly consistent with previously reported mechanistic pathways [52,53,54,55,56,57,58] and aligns well with density functional theory (DFT) studies on metal-catalyzed borohydride hydrolysis [53,54,55]. According to these studies, the hydrolysis reaction proceeds through a surface-mediated, multi-step mechanism involving adsorption, activation, and stepwise dehydrogenation of neutral borohydride species (BH_4_) adsorbed onto metallic Ag sites.

In the first step, both NaBH_4_ and H_2_O molecules physisorb or weakly chemisorb onto the silver surface. The Na^+^ counterion remains solvated, while the neutral BH_4_ species interacts directly with the metallic Ag atoms. The silver sites act as Lewis acid centers, inducing polarization of the B–H bonds and lowering the activation energy required for hydride dissociation.

The first hydrogen molecule evolves through the partial cleavage of a B–H bond, forming a surface-bound Ag–H hydride species and a neutral BH_3_(ad) intermediate that remains associated with the Ag surface. The elementary reaction steps can be summarized as follows:

BH_4_(ad) → BH_3_(ad) + H(ad) (4)

Ag + H(ad) → Ag–H(5)

Ag–H + H(ad) → H_2_↑ + Ag (6)

BH_3_(ad) + 3H_2_O → B(OH)_3_ + 3H_2_↑ (7)

In this mechanism (Equations (4) to (7)), BH_4_(ad) and BH_3_(ad) represent neutral surface-adsorbed intermediates, stabilized through weak Ag–B and Ag–H interactions, as supported by DFT calculations [53,54,55]. The final hydrolysis of BH_3_ produces boric acid (B(OH)_3_) and three additional hydrogen molecules, completing the catalytic cycle. This pathway aligns with the overall stoichiometric reaction for NaBH_4_ hydrolysis (Equation (2)).

Experimental kinetic data, including turnover number (TON = 1.4 × 10^5^–1.9 × 10^5^) and turnover frequency (TOF = 7.1 × 10^4^–9.3 × 10^4^ h^−1^), confirm that the AgSC catalyst possesses abundant and accessible active sites capable of facilitating efficient hydrogen evolution. The pH-dependent results further substantiate this mechanistic interpretation. Under acidic conditions (pH 6), although the intrinsic hydrolysis kinetics are faster due to the higher proton concentration, the overall hydrogen yield decreases. This reduction is attributed to bubble-induced mass transfer limitations, catalyst surface restructuring, and partial deactivation caused by borate by-products. At neutral pH (7), hydrogen generation reaches its maximum value (16.1 mL), indicating an optimal balance between hydrolysis kinetics and catalyst stability. Conversely, under basic conditions (pH 8), stabilization of the BH_4_^−^ anion suppresses hydrolysis, resulting in a lower hydrogen yield (12.8 mL).

These experimental observations, specifically regarding the activation energy (54 kJ·mol^−1^), TON, TOF, pH-dependent performance, and reusability, correlate strongly with mechanistic insights from reported density functional theory (DFT) and spectroscopic studies on NaBH_4_ hydrolysis over metallic surfaces. Literature-based DFT models highlight electron redistribution at the Ag–C interface, stabilization of transition states, and surface-mediated hydride transfer as key factors that enhance reaction kinetics. These phenomena explain how Ag nanoparticles and the carbon microsphere support act synergistically to promote hydrogen generation, lower activation barriers, and stabilize catalytic intermediates [53,54,55,56,57,58].

Reusability experiments further demonstrated the robustness and durability of the AgSC catalyst. After five consecutive NaBH_4_ hydrolysis cycles, AgSC retained more than 95% of its initial catalytic activity, showing only a minor decrease (approximately 4–5%) in hydrogen yield. This minimal loss indicates excellent structural stability, resistance to nanoparticle aggregation, and strong interfacial bonding between silver nanoparticles and the carbon support. The close correspondence between the XRD-derived crystallite size (~14 nm) and the TEM-observed particle size (~12 nm) confirms the high crystallinity and uniform dispersion of active sites, ensuring effective surface accessibility crucial for sustained catalytic performance.

Collectively, these findings establish that AgSC-catalyzed NaBH_4_ hydrolysis proceeds via a surface hydride-mediated mechanism, consistent with reported DFT-supported models of metal-assisted hydrolysis [53,54,55]. The catalytic behavior is strongly influenced by solution pH, while the observed long-term stability is primarily governed by surface fouling effects, such as limited borate deposition through repeated use.

## 4. Conclusions

Silver nanoparticles supported on glucose-derived carbon microspheres (AgSC) were successfully synthesized and evaluated as an efficient catalyst for sodium borohydride (NaBH_4_) hydrolysis to produce hydrogen. Structural characterization by XRD, TEM, SEM, and EDS confirmed the uniform distribution of metallic Ag nanoparticles on the carbon support with no detectable Ag_2_O phase, demonstrating excellent crystallinity and oxidation resistance. The AgSC catalyst outperformed both unsupported Ag nanoparticles and bare carbon, highlighting a strong synergistic effect between the silver and carbon components. Catalytic studies revealed that hydrogen generation strongly depends on reaction conditions. Maximum hydrogen evolution (16.1 mL) occurred at neutral pH (7), where optimal kinetics and catalyst stability were achieved. Acidic conditions (pH 6) showed slightly lower yields (15.1 mL) due to bubble blockage, borate surface poisoning, and partial Ag leaching, while basic media (pH 8) reduced hydrogen production (12.8 mL) due to BH_4_^−^ stabilization. Kinetics analysis determined an activation energy of 54 kJ mol^−1^, with turnover numbers (TONs) of 1.4 × 10^5^–1.9 × 10^5^ and turnover frequencies (TOFs) of 7.1 × 10^4^–9.3 × 10^4^ h^−1^, confirming high catalytic efficiency and active site accessibility. Reusability tests showed only minor activity loss (~4–5%) after five cycles, indicating strong catalyst durability. Collectively, these results establish AgSC as a robust, recyclable, and high-performance catalyst for on-demand hydrogen generation from NaBH_4_, offering a sustainable route toward practical clean fuel technologies and advancing green energy catalyst design.

## Figures and Tables

**Table 1:** Comparison of catalysts used for hydrolysis of NaBH_4_.

Catalyst	E_a_ (kJ mol^−1^)	Temperature (K)	Reference
Silica sulfuric acid	17	298–343	[45]
Ru/Graphite	61.1	398–318	[46]
Co-B	64.9	283–303	[47]
CoCl_2_	17.5	293–308	[48]
MWCNT-supported Co	40.4	298–333	[49]
Ru/C	67	298–358	[50]
Pd/C	28	298–328	[51]
AgSC	54	273–303	This Study

## Data Availability

The original contributions presented in this study are included in the article/Supplementary Materials. Further inquiries can be directed to the corresponding author.

## Supplementary Materials

The following supporting information can be downloaded at https://www.mdpi.com/article/10.3390/ma18214912/s1, Figure S1: SEM-EDX of silver-coated spherical carbon; Figure S2: The hydrogen generated over time with various amounts of the reactant NaBH_4_; Figure S3: The hydrogen generated over two hours under conditions of increased (pH 8) and decreased (pH 6) pH; Figure S4: Reusability of AgSC catalyst for NaBH_4_ hydrolysis; Table S1: Summary of rates, TONs, and TOFs of AgCS catalysts in NaBH_4_ hydrolysis.
