# Supplementary material for: Spherical Carbon Derived from Sustainable Sources and Decorated with Silver Nanoparticles as a Catalyst for Hydrogen Release
Source: Materials (Basel). 2025 Oct 27;18(21):4912. doi: 10.3390/ma18214912 (PMC12609952; doi:10.3390/ma18214912)
Supplement: Supplementary file 1 [file materials-18-04912-s001.zip › materials-3904840-supplementary.pdf]

Supplementary Information

Spherical Carbon Derived from Sustainable Sources and Decorated with Silver Nanoparticles as a Catalyst Material for Hydrogen Release

Erik Biehler and Tarek M. Abdel-Fattah *

Applied Research Center, Thomas Jefferson National Accelerator Facility, Department of Biology, Chemistry and Environmental Science, Christopher Newport University, Newport News, VA 23606, USA;
erik.biehler@cnu.edu
* Correspondence: fattah@cnu.edu

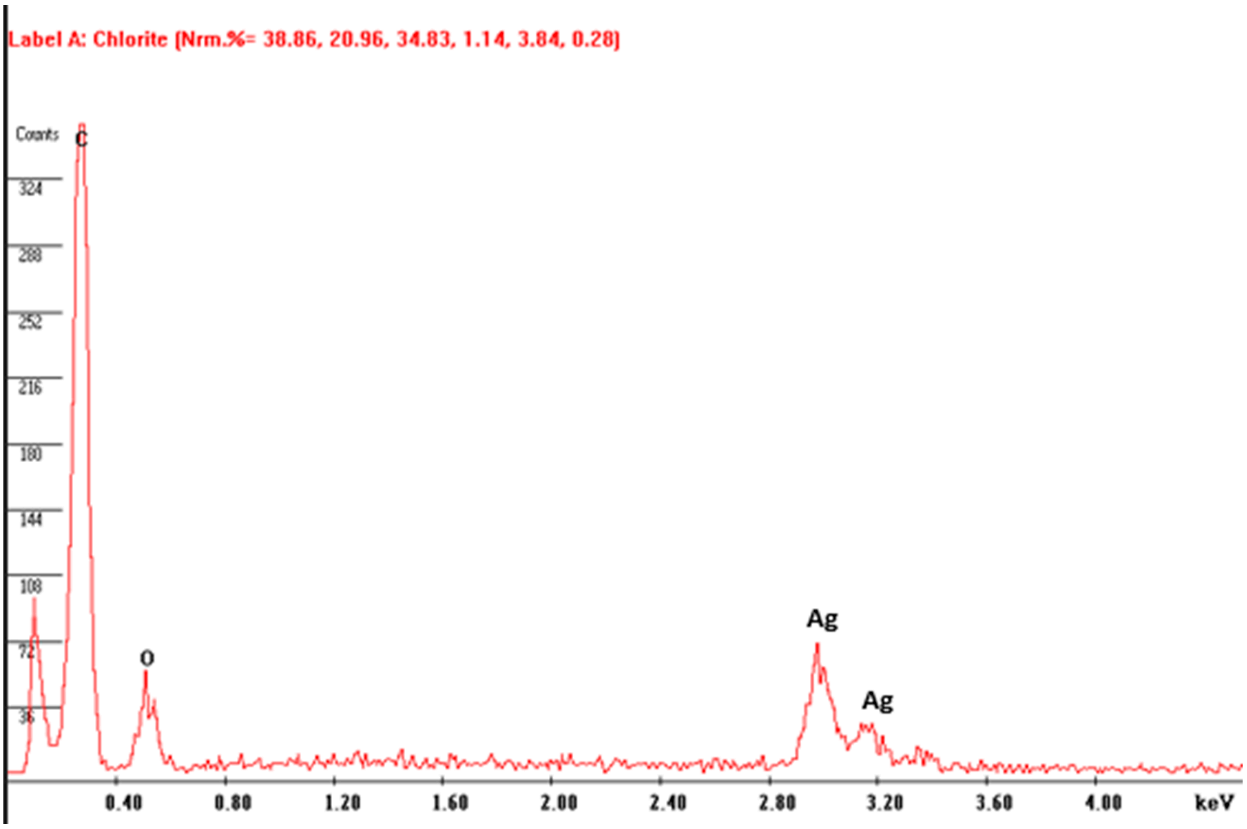

Figure S1. SEM-EDX of silver-coated spherical carbon.

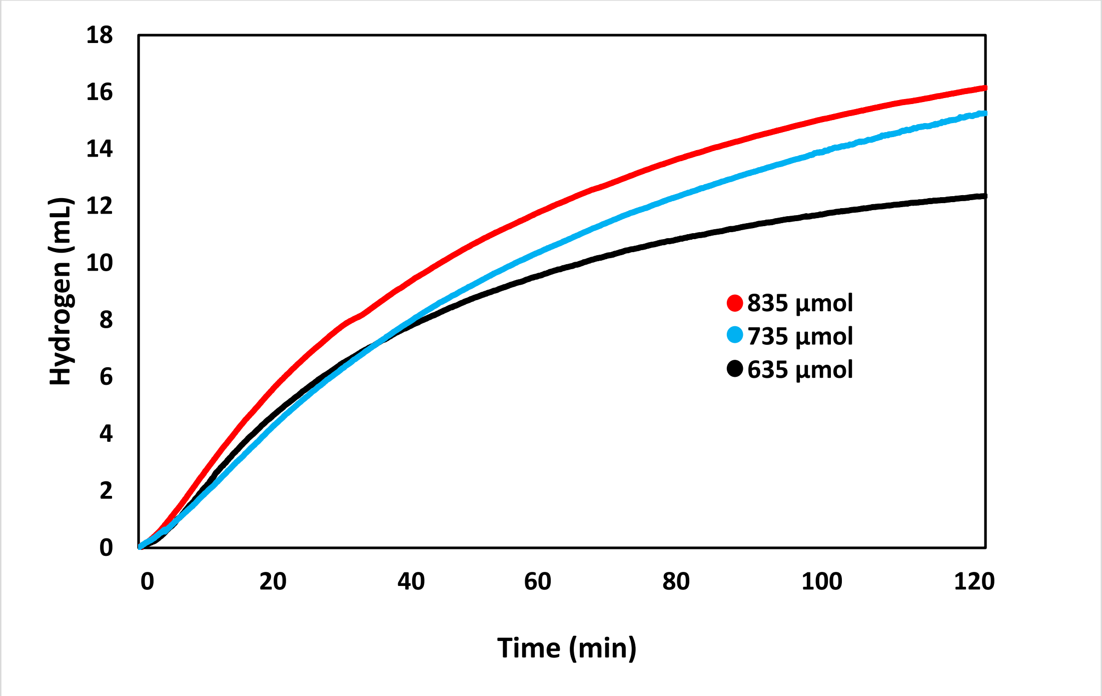

Figure S2. The hydrogen generated over time with various amounts of the reactant $NaBH_4$.

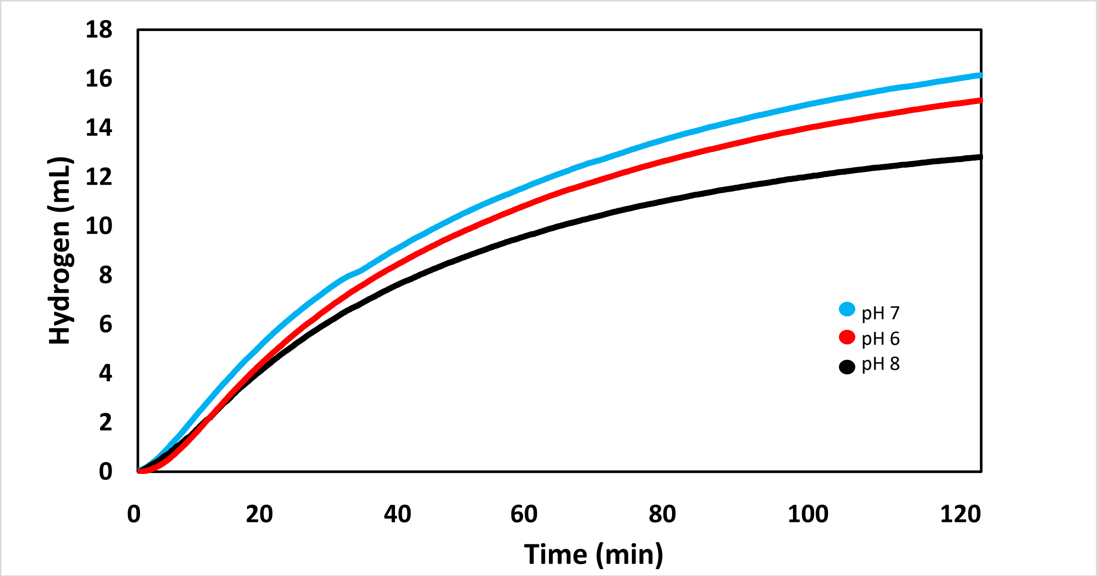

Figure S3. The hydrogen generated over two hours under conditions of increased (pH 8) and decreased (pH 6) pH.

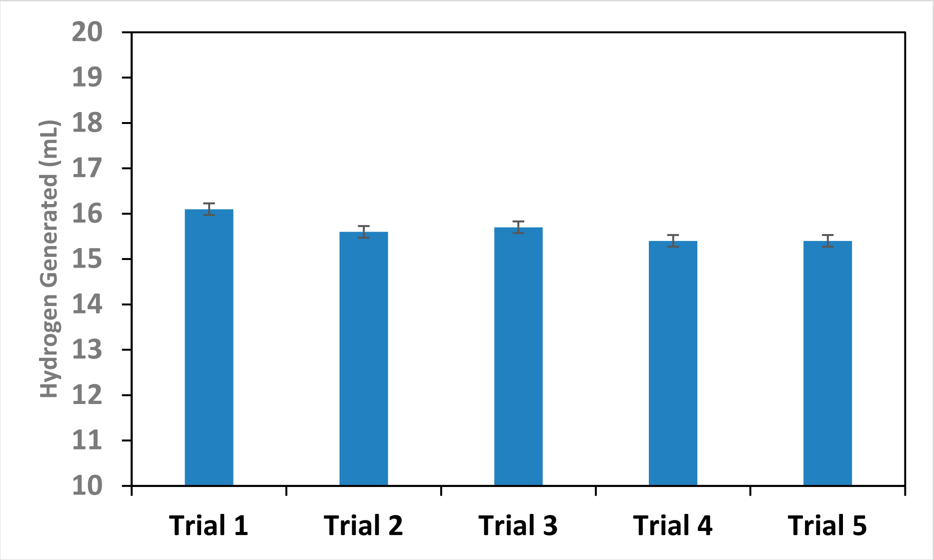

Figure S4. Reusability of AgSC catalyst for $NaBH_4$ hydrolysis.

Table S1. Summary of rates, TONs, and TOFs of AgCS catalysts in $NaBH_4$ hydrolysis.

Case	Rate (mL/min)	Time (min)	H_2 Volume (mL)	H_2 (mol) x 10^{-4}	TON x 10^5	TOF (min^{-1})	TOF (h^{-1})
R1	0.1025	120	12.3	5.488	1.42	1184	71,033
R2	0.1275	120	15.3	6.826	1.77	1473	88,358
R3	0.1342	120	16.1	7.185e	1.86	1550	92,978
